# “My Worries Are Rational, Climate Change Is Not”: Habitual Ecological Worrying Is an Adaptive Response

**DOI:** 10.1371/journal.pone.0074708

**Published:** 2013-09-04

**Authors:** Bas Verplanken, Deborah Roy

**Affiliations:** Department of Psychology, University of Bath, Bath, United Kingdom; The Ohio State University, United States of America

## Abstract

Qualifications such as “global warming hysteria” and “energy policy schizophrenia” put forward by some climate change skeptics, usually outside the academic arena, may suggest that people who seriously worry about the environment suffer from psychological imbalance. The present study aimed to refute this thesis. While habitual worrying in general is strongly associated with psychopathological symptoms, in a survey a near-zero correlation was found between habitual ecological worrying and pathological worry. Instead, habitual ecological worrying was associated with pro-environmental attitudes and behaviors, and with a personality structure characterized by imagination and an appreciation for new ideas. The study had sufficient statistical power and measures were valid and reliable. The results confirm that those who habitually worry about the ecology are not only lacking in any psychopathology, but demonstrate a constructive and adaptive response to a serious problem. In the public domain, these findings may contribute to a more rational and less emotional debate on climate change and to the prevention of stigmatization of people who are genuinely concerned about our habitat and are prepared to do something about it (“habitual worriers are not crazy”). In the academic arena this study may contribute to environmental psychology (“habitual worrying is part of a green identity”), as well as to the literature on worry and anxiety (“habitual worrying can be a constructive response”).

## Introduction

Since the heated debates on nuclear energy, no other environmental issue has triggered such strong emotions as global warming. Self-proclaimed global warming skeptics, who deny the human factor in global warming or global warming itself, accuse scientists, politicians and concerned citizens of biased opinions and bad science. Those who overly worry about climate change are sometimes stigmatized as being mentally unstable or pathological, which is suggested by terms such as “mass neurosis”, “energy policy schizophrenia”, “tree huggers”, or the “global warming hysteria” [1], [2], [3]. Because these ‘debates’ are usually held outside the academic arena and seldom focus on the merits of scientific findings, it is easy to dismiss such qualifications as ludicrous and their advocates as an odd bunch. However, in order to contribute to a more productive, decent, and less emotional debate, we wish to investigate these accusations in more detail in an exploratory study.

It is undoubtedly the case that environmental threats from climate change affect some people’s personal feelings of well-being and may cause stress and anxiety [4], [5], [6], [7], [8], [9], [10]. Specific populations have also good reasons to be worried and anxious, if they are directly exposed to health risks or increased morbidity rates linked to climate-related events, such as flooding, mega storms, heat waves or droughts [11], [12], [13]. However, the vast majority of people who are concerned about climate change are not directly affected, but experience climate change impact vicariously through media exposure to information about risks, devastating events elsewhere, or debates about negative future consequences [14]. One might argue that if these individuals experience high levels of worry and anxiety about climate change, this is not a rational response, and might indeed be a symptom of underlying mental health problems or manifestations of comorbidity of anxiety-related conditions [15]. In this article we aim to dispute this thesis, and instead demonstrate that even high levels of ecological worrying (habitual worrying) are constructive and adaptive, i.e., are associated with pro-environmental attitudes and actions, and are not related to maladaptive forms of worrying such as pathological expressions of anxiety.

### Habitual Worrying

Our mind has the capacity of mental time traveling, which enables us to remember the past and form representations of the future. Worrying is a manifestation of this faculty. Worrying often occurs when we anticipate hypothetical challenges, difficulties or potentially dangerous situations. It is in essence an adaptive response, which enables us to be prepared, find solutions, or recruit resources, all of which are aimed at mitigating expected risks and coping with potential problems [16].

However, when worrying becomes a dominant feature of the mind, and occurs repetitively and persistently, it may become dysfunctional, and may be a symptom of deeper seated pathological conditions, such as generalized anxiety disorder [15], [17], or more specific anxieties such as hypochondria [18], or parental anxiety [19]. Worrying may thus become a *mental habit*, i.e., thinking that occurs frequently and automatically [20]. Habitual worried thinking has been found strongly associated with symptoms of pathological worry and anxiety [17], [21], [22], [23]. Based on these findings, one would expect that individuals who habitually worry about the environment show indications of dysfunctional anxiety-related conditions. This, then, would confirm the alleged psychopathological conditions attributed to ecological worriers, which we referred to in the introduction paragraph.

### Ecological Engagement

While habitual worrying has been found dysfunctional, why then would habitual worrying about the environment be associated with *constructive* outcomes, rather than with psychopathological conditions? Whereas pathological worrying is characterized by detrimental outcomes such as anxiety, negative affect, stress and impaired health, constructive worrying is associated with outcomes such as positive mental health, functional cognitive operations and behaviors (e.g., generating plans, taking initiatives and problem solving), and, under some conditions, better performances [17]. There are several reasons why habitual ecological worrying is constructive rather than unconstructive or pathological. Firstly, although worry and anxiety are often closely linked, there is evidence to suggest that these are separate constructs, each with their own unique sources of variance [24]. Secondly, in a pathological context, worrying is often a response to intrapsychic struggles, for instance involving dysfunctional self-beliefs (e.g., “I will be punished for not controlling my thoughts”) and personality traits (e.g., neuroticism) [25]. Ecological worries, on the other hand, are primarily externally focused. Thirdly, repetitive worrying has been found associated with beneficial outcomes under certain conditions [17]. For instance, repetitive worrying is more likely to be constructive if it is focused on finding solutions and problem solving, rather than mere rumination on problems and negative consequences. Worrying also tends to be constructive if it is accompanied by feelings of personal competence and efficacy, which may lead to finding solutions. These conditions are more likely to prevail among segments of the population which are deeply concerned about the environment, such as those who feel affiliated to the ecological or green movement.

More formally, Stern presented the value-belief-norm (VBN) theory of environmentalism, which he defined as the propensity to take actions with pro-environmental intent [26]. VBN theory proposes that such behaviors are rooted in worries about threats to the biosphere, which in turn are embedded in biospheric and altruistic values and an ecological worldview. Together with beliefs about self-efficacy, these may lead to a felt obligation or personal norm to take action, and to pro-environmental behavior [27]. VBN theory thus typically describes the conditions for constructive worrying. Furthermore, the chronic nature of the underlying biospheric values and worldviews would be a reason why for these individuals ecological worrying has become habitual.

Taking these considerations together, and contrary to what findings of habitual worrying being associated with symptoms of pathological conditions would suggest, it was expected that habitual ecological worrying is constructive and associated with positive attitudes toward the environment (H1) and pro-environmental behaviors (H2). Moreover, a *non-*significant correlation was predicted between habitual ecological worrying and pathological worry (H3). Because the latter hypothesis involved the prediction of a null effect, evidence for the reliability and validity of the measures used to test this hypothesis is paramount. In addition to the internal reliability of the scales, evidence for the validity of the habitual worrying and pathological worry scales was obtained by the number of spontaneously elicited ecological worries and a measure of the Big Five personality traits, respectively.

## Methods

### Participants and Procedure

The study consisted of an online survey, which was posted at university websites in the US and Europe, and ran for three weeks in June/July 2012. A total of 132 participants completed the study. There were 39 men and 78 women, while 15 participants did not disclose their sex. All participants were 18 years or older. The average age was 26 years (*SD* = 10 years; range = 18–67 years). Two participants were high school pupils, 63 participants were undergraduate university students, 47 participants were postgraduate university students, and 20 participants were non-students. 102 participants were located in Europe, 21 in North America, and 9 elsewhere.

The survey contained assessments of ecological worrying, environmental attitudes, prevalence of pro-environmental behaviors, pathological worrying, and the Big Five personality traits. At the end of the survey participants were provided with the opportunity to submit any comments.

#### Ethics statement

The study received full approval from the Departmental Ethics Committee, Department of Psychology, University of Bath (reference number 12-070). In the introduction of the study participants were explicitly told that informed consent was assumed if they continued and submitted their data.

### Measures

### Habitual ecological worrying

The study was introduced as follows: “In this study we want to know more about worries people may have about the natural environment. This is not restricted to the area you live, but may refer to any place or area and any aspect of nature or natural events, including the earth itself.” This was followed by the question “How often do you have thoughts about the environment, which you find worrying, uncomfortable, or upsetting?”. Response categories were “never”, “every now and then”, “sometimes”, “often”, and “all the time”. Participants who ticked one of the latter four responses (*N = *120) were then presented with a thought-listing task, in which they wrote down the worries they sometimes have. Space was provided to enter a maximum of ten worries. This was followed by the twelve-item Habit Index of Negative Thinking [20], which in the present case was adapted to assess habitual worried thinking. The instruction referred to the worries that were elicited in the thought-listing task. Each item starts with the stem “Having those worrying thoughts is something …”. Sample items are: “I do frequently”; “I find hard not to do”; “I start doing before I realize it”. Responses were given on five-point scales (strongly disagree - strongly agree), *alpha* = 0.97. The items were averaged. High scores indicate a strong habit of worrying.

#### Environmental attitudes

Attitudes toward the environment were assessed by the twenty-four item Environmental Attitude Inventory [28]. Sample items are “It makes me sad to see forests cleared for agriculture”, “Humans are severely abusing the environment”, and “Protecting people’s jobs is more important than protecting the environment” (reverse-coded). Responses were given on five-point scales (strongly disagree - strongly agree), *alpha* = 0.82. The items were averaged. High scores indicate a positive attitude or a strong concern.

#### Pro-environmental behavior

Participants were presented with sixteen pro-environmental behaviors, and indicated how often they had performed these activities in the last year. The following behaviors were included: taking shorter showers; switching off the water tap while brushing your teeth; switching off electrical appliances instead of leaving them on standby; taking a used or reusable shopping bag when shopping; switching off lights when leaving a room; disposing garbage in the proper recycling bins or bags; using other modes of transportation than the car (for car owners only); buying organic products; buying locally produced products; buying a less polluting product when given the choice; switching off the heating on time before going out or to sleep; making sure to maintain a comfortable, but not higher than strictly necessary, temperature in room or house; monitoring electricity and/or gas consumption; making sure not to spoil gas or electricity while cooking; talking about environmental issues with others; being a member of an environmental organization. Responses were given on five-point scales, labeled by “never”, “seldom”, “sometimes”, “often”, and “always”, respectively, *alpha* = 0.84. The items were averaged. High scores indicate higher frequencies of pro-environmental behavior.

#### Pathological worry

Symptoms of pathological worry were assessed by the sixteen items Penn State Worry Questionnaire [29]. This is a widely accepted and validated instrument, which, for example, discriminates samples that meet diagnostic criteria for generalized anxiety disorder. Sample items are “My worries overwhelm me”, “Many situations make me worry”, and “I worry all the time”. Responses were given on five-point scales (strongly disagree - strongly agree), *alpha* = 0.93. The items were averaged. High scores indicate higher levels of pathological worry.

#### Big Five personality traits

An indication of participants’ Big Five personality trait profile was obtained by a ten item short version of the Big Five Inventory [30]. Each of the Big Five traits was assessed by two items. The correlations between the item pairs for Extraversion, Agreeableness, Conscientiousness, Emotional Stability, and Openness were 0.58, *p*<.001; 0.35, *p*<.001; 0.45, *p*<.001; 0.55, *p*<.001, and 0.26, *p*<.01, respectively. The respective items for each trait were averaged.

### Statistical Analyses

The statistical analyses consisted of bivariate correlations and confidence intervals for key correlations.

## Results

Twelve participants (9%) indicated they never had worrying thoughts about the environment. For “every now and then”, “sometimes”, “often” and “all the time” these numbers were 26 (20%), 42 (32%), 32 (24%), and 20 (15%), respectively (*M* = 3.17, *SD* = 1.18). For obvious reasons, participants who indicated they never had worrying thoughts about the environment did not list worried thoughts and had missing values on the measure of habitual ecological worrying. The remaining 120 participants generated a total of 660 worries, on average 5.50 per participant (*SD = *2.65), or 5.00 per participant (*SD = *2.99) across all 132 participants. In order to investigate the content of the worries, these were grouped into twelve categories. The prevalence of the worries generated in each category, as well the number of worries in each category if these appeared as first, second or third in the participant’s thought-list, are presented in Table 1. As can be seen, global warming, pollution, extinction of species, resource depletion, and deforestation were the five most prevalent worries. As far as could be inferred from the protocols, most if not all ecological worries were focused on the future. This perhaps differentiates ecological worries from worrying in general, which, although future orientations dominate, is characterized by a mixture of thoughts about the future, present and past [16].

**Table 1:** Worries generated in the thought-listing task.

		*N* of worries	*N* of worries in first three worries elicited
1	Global warming, climate change	124	77
2	Pollution, environmental damage	113	61
3	Extinction of species, biodiversity	84	41
4	Resource depletion, lack of renewables	76	47
5	Deforestation, desertification	44	23
6	Waste, landfill	38	23
7	Overpopulation, urbanization	32	16
8	Food shortages, health problems	28	7
9	Own or other peoples’ behaviors	22	10
10	Insufficient recycling	20	13
11	Economy, politics	18	9
12	Miscellaneous	61	15
	Total number of worries generated	660	342

The distributions of the study variables were normal. Measures of skewness and kurtosis were within +/−1, with the exception of pro-environmental behavior (skewness = −1.06, kurtosis = 2.37) and Openness (kurtosis = −1.18). In Table 2 means, standard deviations and Pearson intercorrelations of all study variables are presented. Inspection of non-parametric correlations for pro-environmental behavior and Openness yielded identical conclusions.

**Table 2:** Means, standard deviations, and correlations between the study variables.

	*M (SD)*	2	3	4	5	6	7	8	9	10
1. Habitual ecological worrying	3.24 (0.83)	0.43***	0.47***	0.37***	−0.05	0.03	−0.20*	0.15	0.16	0.22*
2. Number of worries	5.00 (2.99)		0.41***	0.47***	−0.04	0.06	−0.06	0.17	0.17	0.30***
3. Environmental attitude	3.80 (0.44)			0.27***	−0.12	0.09	−0.13	0.05	0.17	0.26**
4. Pro-environmental behaviors	3.71 (0.68)				0.03	0.11	0.22*	0.26**	0.18*	0.10
5. Pathological worry	3.10 (0.82)					−0.29**	−0.22*	−0.17	−0.73***	−0.05
6. Extraversion	3.10 (1.01)						0.20*	0.15	0.35***	0.08
7. Agreeableness	3.63 (0.87)							−0.03	0.18*	−0.14
8. Conscientiousness	3.84 (0.87)								0.27**	0.13
9. Emotional stability	3.00 (1.10)									0.16
10. Openness	3.79 (0.92)									

Notes: *N* = 132 (*N* = 120 for correlations with habitual ecological worrying); * = *p*<.05; ** = *p*<.01; *** = *p*<.001.

The correlations indicate that all three hypotheses were supported. Habitual ecological worrying correlated moderately strongly with environmental attitudes, *r* = 0.47; *p*<.001; 95% confidence interval: +0.32 to +0.60, and pro-environmental behavior *r* = 0.37; *p*<.001; 95% confidence interval: +0.20 to +0.52. Note the positive signs of these correlations: habitual ecological worrying is associated with positive environmental attitudes and a prevalence of pro-environmental behaviors. There were no statistically significant relationships between pathological worry and the ecology-related variables, including, importantly, habitual ecological worrying. As expected, the correlation between habitual ecological worrying and pathological worry was close to zero, *r* = −0.05, *p = *0.59; 95% confidence interval: −0.23 to +0.13.

Because H3 comprised a null effect, it is important to have confidence in the reliability and validity of the measures. As reported above, the internal reliabilities of the measures of habitual ecological worrying and pathological worry were excellent (*alphas* >0.90). Support for the validity of the habit scale can be obtained from the correlation with the number of spontaneously elicited ecological worries, *r* = 0.43, *p*<.001; 95% confidence interval: +0.27 to +0.57, which is in the range of correlations that are found when using this paradigm [20], [23]. The validity of the pathological worry scale was supported by the correlations with the Big Five personality traits, in particular the strong correlation with Emotional Stability, *r* = −0.73, *p*<.001; 95% confidence interval: −0.80 to −0.63.

Table 2 shows a number of other interesting correlations associated with the personality traits. The number of ecological worries, the habitual quality of ecological worrying, and environmental attitudes correlated weakly but significantly and positively with Openness. Pro-environmental behaviour correlated positively with Agreeableness, Conscientiousness, and Emotional Stability. Taken together, these correlations support the notion that worries about the environment and pro-environmental attitudes are associated with positive mental health and well-being.

## Discussion

The quote in the title is from one of the participants in our study, and summarizes the message of this article: habitual ecological worrying is a constructive and adaptive response to a serious problem. Whereas habitual worrying in other contexts is associated with pathological mental conditions, such as generalized anxiety disorder, in the present study a near-zero correlation between habitual ecological worrying and pathological worry was expected and found. The adaptive nature of habitual ecological worrying was demonstrated by the correlations with positive environmental attitudes and pro-environmental behaviors, and (weaker but statistically significant) with Openness as one of the Big Five personality traits. This trait involves a set of qualities such as imagination, aesthetic sensitivity, preference for variety, and intellectual curiosity. The pro-environmental attitudes and behaviors can be interpreted as manifestations of the potentially adaptive qualities of worrying, which involve taking initiatives and attempts at problem solving. The personality constellation thus provides a structural psychological background fostering such responses. Taken together, contrary to what some have suggested in the debates around this topic, people who habitually worry about the ecology are not only lacking in any form of mental instability, but seem genuinely concerned, show engagement with the green agenda, have a motivation to act accordingly, and are characterized by an open mind.

Opinion polls consistently suggest that the segment of the population which is genuinely concerned about the ecology is very small indeed. And, if measured by the lack of effective action by countries and international platforms, this also holds for politicians. While people happily express the opinion that protecting the ecology is “important”, this topic usually ends up as completely *un*important when compared to a host of other issues [31]. Also, structural and psychological barriers exist which jeopardize effective pro-environmental action [32]. And even if people act pro-environmentally, behaviors may not always be the ones with the highest impact [33]. However, the ecological or green movement demonstrates that genuinely concerned individuals do exist. In the present study those with high scores on habitual ecological worrying may well represent these individuals. From the literature on environmentalism and ecological concern a profile of this segment emerges as individuals who endorse biospheric and altruistic values [26], [34], [35], [36], hold an ecological worldview [37], believe that the ecology is threatened, but also that they can be effective in mitigating these threats, and have pro-environmental personal norms and habits [38], [39], [40]. In addition, collective beliefs may be involved, such as feelings of collective guilt [41] and collective efficacy [42]. An important feature of the genuinely concerned segment is that ecology-related values and attitudes are part of individuals’ self-concept or an ecological or ‘green’ identity [43], [44], [45], [46], [47].

The present discussion may raise some conceptual issues relating to the distinction between “worry” and “concern”. In a general sense, worry refers primarily to an emotional reaction (albeit with a cognitive content), while concern has a more rational tone, and in the present context can be interpreted as an attitude toward or the perception of risk [39]. Sjöberg showed that worry and the perceptions of risks were only weakly correlated [48]. While worry and concern are thus distinct concepts, it may well be that for the “genuinely concerned” individuals this distinction is less obvious.

As alluded to before, hypothesizing and finding null effects is treacherous. In this case, we think the near-zero correlation between habitual ecological worrying and pathological worry is insightful and meaningful. Nevertheless, it is imperative to consider potential alternative explanations or flaws, which might lead to observing a non-significant correlation. Three obvious candidates are lack of power, unreliability or invalidity of the measures, and the quality of the data collection. As for statistical power, the effective sample size of 120 (i.e., when involving habitual ecological worrying) would detect a moderate correlation of 0.25, with alpha set at 0.05 and a power of 0.80. Correlations between measures of habitual thinking and psychopathological symptoms are typically found to be in the 0.40 - 0.50 range [20], [21], [22], [23]. In other words, this power analysis suggests that a sample size of only approximately 40 would have been required to reliably detect correlations of such magnitudes. It can thus be safely concluded that the study had sufficient statistical power to identify a significant relationship between habitual ecological worrying and pathological worry, if there was any. Secondly, we can have confidence that the psychometric qualities of instruments used to test H3 were up to standard; the internal reliabilities were excellent, and while both instruments have been used and validated in previous research, the present study provided additional validating support in the form of significant correlations between habitual ecological worry and the number of worries elicited, and between pathological worry and emotional instability, respectively. Finally, as for confidence in the quality of the data collection, the data were scrutinized for careless responding and participants were only included if they had fully completed the online study.

The study has limitations due to the nature of the sample (university students), which clearly limits the generalizability of the conclusions to larger populations. The method of data collection (online survey) might also pose some limitations. However, Gosling and colleagues demonstrated that there is no reason to suggest that these methods provide less quality data than traditional methods [49]. Another potential limitation might be posed by demand characteristics, such as the tendency to respond in a socially desirable direction or to respond consistently. While such biases may have had some effect on the size of the correlations between the environment-related variables, these cannot easily explain the near-zero correlation between habitual ecological and pathological worrying. Finally, the study might have benefited from the inclusion of a more varied set of psychopathology indicators. While the Penn State Worry Questionnaire covered anxiety-related conditions, other indicators such as of paranoia would have been interesting as well.

We consider this study as a ‘proof of concept’. By identifying the existence of a healthy form of worrying in the environmental domain the study may contribute to a more rational debate on climate change and to prevention of stigmatization of people who are genuinely concerned about our habitat and are prepared to do something about it (“habitual worriers are not crazy”). This study may also contribute to the domain of environmental psychology by suggesting that habitual ecological worrying is a defining characteristic of those who are genuinely concerned (“habitual worrying is part of a green identity”). Finally, this study adds another example of constructive repetitive worrying to the literature on worry and anxiety (“habitual worrying can be a constructive response”).
